# Emerging roles of i-motif in gene expression and disease treatment

**DOI:** 10.3389/fphar.2023.1136251

**Published:** 2023-03-20

**Authors:** Xiaoqing Luo, Jianye Zhang, Yue Gao, Weifei Pan, Yayuan Yang, Xu Li, Lingfei Chen, Chang Wang, Yuqing Wang

**Affiliations:** Guangzhou Municipal and Guangdong Provincial Key Laboratory of Molecular Target and Clinical Pharmacology, The NMPA and State Key Laboratory of Respiratory Disease, School of Pharmaceutical Sciences and the Fifth Affiliated Hospital, Guangzhou Medical University, Guangzhou, China

**Keywords:** i-motif, biological activities, molecular mechanism, ligand compounds, oncogene, telomere, gene-targeted therapy

## Abstract

As non-canonical nucleic acid secondary structures consisting of cytosine-rich nucleic acids, i-motifs can form under certain conditions. Several i-motif sequences have been identified in the human genome and play important roles in biological regulatory functions. Due to their physicochemical properties, these i-motif structures have attracted attention and are new targets for drug development. Herein, we reviewed the characteristics and mechanisms of i-motifs located in gene promoters (including *c-myc*, *Bcl-2*, *VEGF*, and telomeres), summarized various small molecule ligands that interact with them, and the possible binding modes between ligands and i-motifs, and described their effects on gene expression. Furthermore, we discussed diseases closely associated with i-motifs. Among these, cancer is closely associated with i-motifs since i-motifs can form in some regions of most oncogenes. Finally, we introduced recent advances in the applications of i-motifs in multiple areas.

## 1 Introduction

DNA carries the genetic information necessary to synthesize RNA and proteins, which are essential for the development and maintaining the normal function of living organisms. Scientists have never stopped exploring the function and application of DNA. In 1953, Watson and Crick (1974) first put forward the molecular model of DNA double helix structure, namely, B-form DNA (B-DNA), which describes the DNA molecule as a right-handed twisted coil composed of a purine and pyrimidine inner core held together by hydrogen bonds, with a sugar-phosphate backbone extending from these paired bases. Further studies have revealed the conformation of A-DNA, Z-DNA, triplex DNA, hairpins, and cruciform, as well as tetraplex structures, including G4 (guanine-quadruplex) and i-motifs (intercalated-motif) (Dickerson et al., 1982; Shakked et al., 1983; Bochman et al., 2012). B-DNA is the major conformation of DNA in the physiological state, while the other configurations are uniformly referred to as non-B-form secondary structures (Bacolla and Wells, 2004) and have not been studied well compared to B-DNA. Therefore, researchers have placed particular emphasis on exploiting treatment strategies in connection with non-B-form secondary structures.

**FIGURE 1:**
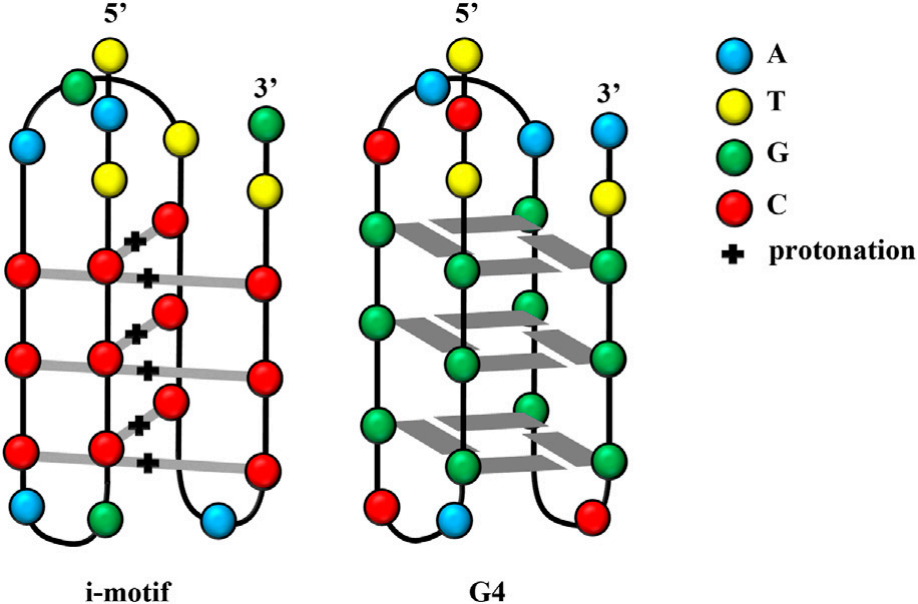
Structures of the i-motif and G4.

In recent decades, G4 structures have attracted increasing research interest. G4 is a non-B-form secondary structure with G-rich nucleic acid sequences under physiological pH. In G4s (guanine-quadruplexes), each guanine interacts with its two adjacent guanines to form a square planar configuration comprising four guanine residues (Figure 1). G4s may occur in one or several different ssDNA or even RNA molecules. Moreover, the extension direction between DNA strands can not only be the same but the opposite; thus, G4s play different roles in gene regulation (Burge et al., 2006; Pascale et al., 2006; Bochman et al., 2012). Thus, G4s are not only DNA sequences that block transcription (Robinson et al., 2021) but can also affect the activity and function of DNA repair (Linke et al., 2021). G4s may also be involved in the development of cutting-edge therapeutic strategies for the treatment of viral infections and cancer (Ruggiero et al., 2021).

Scientists have also discovered another DNA structure with gene regulation function, which follows the principle of complementary base pairing and is present in the complementary cytosine (C)-rich sequences in nucleic acid sequences forming G4s. These C-rich sequences form another type of non-B-form secondary structure known as an i-motif, which forms in slightly acidic microenvironments. Studies have reported that the influential factors of i-motif formation are similar to those of G4 structures. This review summarized researches on i-motifs, including their structure, biological function, mechanism, and application.

I-motif structures are four-stranded non-covalent complexes that form within certain C-rich sequences, where hemi-protonated cytosine-cytosine base pairs intercalate with each other (Day et al., 2014; Mir et al., 2017; Kretzmann et al., 2021; Serrano-Chacón et al., 2021) (Figure 1). These structures can form in both DNA and RNA (Collin and Gehring, 1998) and include intramolecular and intermolecular i-motif structures. The presence of i-motif structures was proven by simulating the normal human physiological state *in vitro* through circular dichroism and native gel electrophoresis (Wright et al., 2017). In 2018, scientists at the Garvan Medical Research Institute in Australia demonstrated the presence of i-motif structures in human cells using fluorescent antibodies against i-motifs, namely, iMabs, which specifically bind to i-motifs (Zeraati et al., 2018).

Huppert and Balasubramanian (2005) suggested that the human genome contains up to 3,776,000 DNA fragments forming G4s; thus, a considerable number of i-motifs are theoretically present. The promoter regions of oncogenes including *Rb* (Jakubowska et al., 2001; Xu and Sugiyama, 2005; Xu and Sugiyama, 2006), *RET* (Guo et al., 2007; Bielecka and Juskowiak, 2015), *VEGF* (Guo et al., 2008; Kimura et al., 2022), *c-myb* (Li et al., 2016), *c-myc* (Sun and Hurley, 2009; Reilly et al., 2014), and *Bcl-2* (Panasiuk et al., 2006; Kendrick et al., 2009; Lin et al., 2013; Yang et al., 2020) contain i-motifs. In addition to those cancer-associated genomic regions, i-motifs also occur in telomeres, centromeres, and ribosomes (Bochman et al., 2012; Mondal et al., 2019).

**FIGURE 2:**
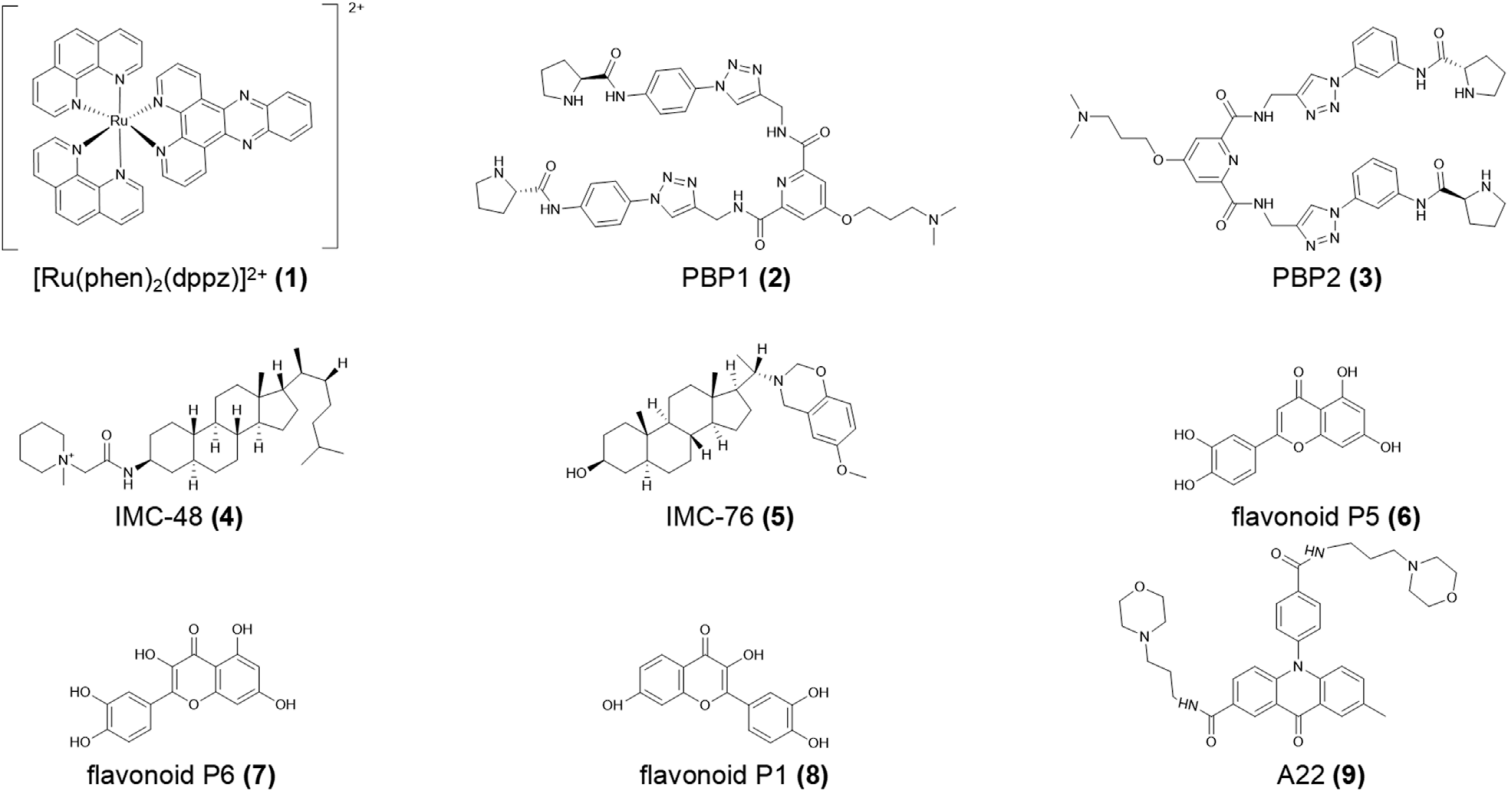
Structures of compounds (**1–9**).

The stabilizing ability of i-motif DNA conformations is also influenced by factors similar to those of G4 structures. The structural stability of G4 depends on many elements, including pH, the specific gene sequence, the size of rings between adjacent bases, temperature, cations, and small molecule ligands (Hardin et al., 2000; Bugaut and Balasubramanian, 2008; Sun and Hurley, 2009; Guédin et al., 2010; Abdelhamid et al., 2019). Many studies have reported factors affecting i-motif structural stability. I-motifs are more sensitive to pH compared to G4s and can fold/unfold swiftly with changes in environmental pH (Del Toro et al., 2009; Jin et al., 2009; Bucek et al., 2010; Lannes et al., 2015; Abou Assi et al., 2018). Moreover, the thermal stability of i-motifs depends on the length of the loop (Reilly et al., 2015); for example, **[Ru**(**phen)**_**2**_ **(dppz)]** ^**2+**^ (**1,** Figure 2) tends to interact with i-motif DNA with longer-length thymidine rings and stabilizes the structure (Pages et al., 2019). Cations such as Na^+^ and K^+^ tend to stabilize/destabilize i-motif structures in different buffer systems (Mathur et al., 2004; Gao and Hou, 2021). Some specific small molecule ligands can combine and stabilize i-motifs (Fedoroff et al., 2000). These effects are summarized in Table 1.

**TABLE 1:** Summary of some properties of i-motifs ligands.

Ligand	Gene/DNA sequence	Effect on the i-motif	Constant	References
**[Ru (phen)2 (dppz)]2+ (1)**	C3TX, e.g., C3T4; C3TXXX, e.g., C3T383	Stabilize	-	Pages et al. (2019)
**PBP1 (2)**	*Bcl-2*	Stabilize	*Kd* = 0.3 μM (pH 4.8) (Fluorescence Titration)	Debnath et al. (2017)
	*c-myc*	Stabilize	*Kd* = 2.4 μM (pH 4.8) (Fluorescence Titration)	
**PBP2 (3)**	*Bcl-2*	Slight	*Kd* = 5.8 μM (pH 4.8) (Fluorescence Titration)	
	*c-myc*	Slight	*Kd* = 9.5 μM (pH 4.8) (Fluorescence Titration)	
**Flavonoid P5 (4)**	*Bcl-2*	-	*K* = (1.1 ± 0.1) × 104 M−1 (ESI);	Yang et al. (2020)
			*K* = (2.8 ± 0.1) × 104 M−1 (PACE-FA)	
**Flavonoid P6 (5)**	*Bcl-2*	-	*K* = (1.4 ± 0.1) × 104 M−1 (ESI);	
			*K* = (2.0 ± 0.1) × 104 M−1 (PACE-FA)	
**Flavonoid P1 (6)**	*Bcl-2*	-	*K* = (1.1 ± 0.1) × 104 M−1 (ESI);	
			*K* = (8.7 ± 0.0) × 103 M−1 (PACE-FA)	
**A22 (7)**	*Bcl-2*	Stabilization	*K*D = 3.56 μM (pH 5.5) (SPR)	Li et al. (2020)
**IMC-76 (8)**	*Bcl-2*	Destabilization	*K*d = 1.01 μM (pH 5.9) (FRET Assay)	Kendrick et al. (2014)
**IMC-48 (9)**	*Bcl-2*	Stabilization	*K*d = 0.49 μM (pH 6.3) (FRET Assay)	
**Fangchinoline (10)**	*c-myb*	-	Ira = 0.27	Li et al. (2016)
**(E)-1-(4-methoxyphenyl)-3-(4-morpholino-6-nitroquinolin-2-yl) prop-2-en-1-one (11)**	*c-myc*	Stabilization	*K*D (compound to hnRNP K) = 4.6 μM (SPR);	Shu et al. (2019)
			*K*D (compound to hnRNP K) = 2.6 μM (MST)	
**TMPyP4 (12)**	Telomere (C3TA2)	Stabilization	Stability constant = 10 (11.8 ± 0.1) M−1 (pH 7.0) (CD)	Fernández et al. (2011)
	Telomere	Stabilization	Stability constant = 10 (12.0 ± 0.1) M−1 (pH 7.0) (CD)	
	(C3TT2)			
**A9 (13)**	*c-myc*	Stabilization	*K*D = 4.5 μM (UV);	Kuang et al. (2020)
			*K*D = 2.4 μM (SPR)	
**ActD (14)**	*c-myc*	Stabilization	*K*b = 9.3 × 104 M-1 (pH 5) (Fluorescence Titration)	Niknezhad et al. (2016)
**Compound 1 (15)**	*HRAS*	Slight	*K*D = 7.4 ± 5.3 μM (pH 6) (SPR)	Journey et al. (2018)
**Compound 2 (16)**	*HRAS*	Slight	*K*D = 5.9 ± 3.7 μM (pH 6) (SPR)	
**Nitidine (17)**	*KRAS*	Destabilization	-	Kaiser et al. (2017)
**NSC309874 (18)**	*PDGFR*	Stabilization	-	Brown et al. (2017)
**B19 (19)**	*c-myc*	Stabilization	*K*D = 7.8 μM (SPR);	Shu et al. (2018)
			*K*D = 4.6 μM (MST)	
**WZZ02 (20)**	*PDGFR*	Destabilization	*K*D = 3.12 μM (SPR)	Zhang et al. (2021)
			*K*D = 4.52 ± 0.488 μM (MST)	
**Fis (21)**	*VEGF*	Destabilization	*K*a = 9.8 × 104 M-1 (pH 5.4) (Fluorescence Titration)	Takahashi et al. (2020)
**BRACO-19 (22)**	Telomere	Destabilization	DC50 = 0.66 μM (pH 4.3) (FID)	Pagano et al. (2018)
**Mitoxantrone (23)**	Telomere	Destabilization	DC50 = 0.70 μM (pH 4.3) (FID)	Pagano et al. (2018)
	Telomere	Stabilization	*K*d = 12 ± 3 μM (pH 5.5) (SPR)	Wright et al. (2016)
	*c-myc*	Stabilization	*K*d = 12 ± 3 μM (pH 5.5) (SPR)	
	Telomere	-	DC50 = 1.8 μM (pH 5.5) (FID)	Sheng et al. (2017)
**Phen-DC3 (24)**	Telomere	Destabilization	DC50 = 0.97 μM (pH 4.3) (FID)	Pagano et al. (2018)
**Pyridostatin (25)**	Telomere	None	DC50 = 9.09 μM (pH 4.3) (FID)	
**RHPS4 (26)**	Telomere	None	-	
**[Tb2 (DL-Cys)4(H2O)8] Cl2 (27)**	Telomere	Slight	*K*b = 4.6 × 104 (pH 7.1) (fluorescence titration)	Xu et al. (2006)
**[Tb2 (DL-HVal)4(H2O)8] Cl6·2H2O (28)**	Telomere	Slight	*K*b = 3.3 × 104 (pH 7.1) (fluorescence titration)	
**Ruthenium (II) polypyridine compound (29)**	-	-	-	Spence et al. (2020)
**Cis-ruthenium (II) polypyridine compound (30)**	Telomere	Stabilization	*K*b = (1.13 ± 0.15) × 106 M-1 (pH 5.5) (absorption titration)	
	DAP	Stabilization	*K*b = (6.94 ± 0.26) × 106 M-1 (pH 6.8) (absorption titration)	
**Mer-ruthenium (II) polypyridine compound (31)**	Telomere	Stabilization	*K*b = 0.63 × 106 M-1 (pH 5.5) (absorption titration)	
	DAP	Stabilization	*K*b = (0.60 ± 0.01) × 106 M-1 (pH 6.8) (absorption titration)	
**Trans-ruthenium (II) polypyridine compound (32)**	Telomere	Stabilization	*K*b = (2.27 ± 0.43) × 106 M-1 (pH 5.5) (absorption titration)	
	DAP	Stabilization	*K*b = (3.39 ± 1.40) × 106 M-1 (pH 6.8) (absorption titration)	
**TO (33)**	*c-myc*; telomere	Stabilization	-	Sheng et al. (2017)
**Tobramycin (34)**	*c-myc*	-	*K*d = 13 ± 1.8 μM (pH 5.5) (SPR)	
	Telomere	-	*K*d = 17 ± 2.0 μM (pH 5.5) (SPR);	
			DC50 = 2.9 μM (pH 5.5) (FID)	
**Alexidine (35)**	Telomere	-	-	
**Tilorone (36)**	Telomere	-	DC50 = 2.4 μM (pH 5.5) (FID)	
**Chlorhexidine (37)**	Telomere	-	-	
**Phenazopyridine (38)**	Telomere	-	-	
**Amodiaquine (39)**	Telomere	-	-	
**Harmalol (40)**	Telomere	-	-	
**Quinalizarin (41)**	Telomere	-	-	
**Minocycline Tyrothricin (42)**	Telomere	-	DC50 > 5 μM (pH 5.5) (FID)	
**[Ru (bpy)2 (dppz)]2+ (43)**	22^−ΔΔCT^	Slight	*K*b = 1.8 × 105 M-1 (pH 5.5; K^+^) (absorption titration)	Shi et al. (2010)
			*K*b = 1.7 × 105 M-1 (pH 5.5; Na^+^) (absorption titration)	
**Berberine (44)**	Telomere	None	DC50 = 30.38 μM (pH 4.3) (FID)	Pagano et al. (2018)
	Telomere	Slight	*K*d = 19.6 μM (pH 6.01) (fluorescence titration)	Xu et al. (2016)
**Phenanthroline compounds 1 (45)**	Telomere	Stabilization	*K*b = (2.3 ± 0.10) × 105 M-1 (pH 5.5) (absorption titration)	Wang et al. (2013)
**Phenanthroline compounds 2 (46)**	Telomere	Stabilization	*K*b = (1.6 ± 0.19) × 105 M-1 (pH 5.5) (absorption titration)	
**Phenanthroline compounds 3 (47)**	Telomere	Stabilization	*K*b = (1.3 ± 0.20) × 105 M-1 (pH 5.5) (absorption titration)	
**1,8-Disubstituted anthraquinone monomer (48)**	Telomere	Stabilization	-	Gouda et al. (2017)
**1,4-Disubstituted anthraquinone monomer (49)**	Telomere	Stabilization	-	
**1,5-Disubstituted anthraquinone monomer (50)**	Telomere	Stabilization	-	
**2,6-Disubstituted anthraquinone monomer (51)**	Telomere	Stabilization	-	
**Terpyridine derivative 1 (52)**	Telomere	Stabilization	*K*b = 1.56 × 106 L/mol (pH 7.4) (absorption titration)	Wei and Gao (2015)
**Terpyridine derivative 2 (53)**	Telomere	Destabilization	-	
**Terpyridine derivative 3 (54)**	Telomere	Destabilization	-	
**BisA (55)**	Telomere	None	-	Bonnet et al. (2022)
**Putrescine (56)**	C6T i-motif	Stabilization	*K*d = 5.3 ± 1.1 mM (CD)	Molnar et al. (2019)
**Spermidine (57)**	C6T i-motif	Stabilization	*K*d1 = 0.003 ± 0.001 mM; Kd2 = 1.0 ± 0.92 mM (CD)	
**Spermine (58)**	C6T i-motif	Stabilization	*K*d = 0.018 ± 0.003 mM (CD)	
**CoII (Chro)2 (59)**	d (CCG)_3_	Destabilization	*K*a = 9.12 × 104 M-1 (pH 7.3) (SPR)	Chen et al. (2018)
	d (CCG)_4_	Destabilization	*K*a = 1.43 × 105 M-1 (pH 7.3) (SPR)	
**Tam (60)**	Telomere	Tam stabilization by interaction with i-motif	-	Heydari et al. (2016)
**Thioflavin T (61)**	*RET*	-	*K*a = 2.516 × 105 M-1 (pH 8.0) (fluorescence titration)	Lee et al. (2015)
	*RB*	-	*K*a = 1.332 × 105 M-1 (pH 5.0) (fluorescence titration)	

^a^ *K*_d_, is the dissociation constant. When the compound has two binding sites, there will have two *K*_d_ values, namely *K*_d1_ and *K*_d2_.

^b^ *K* is the binding constant.

^c^ PACE-FA means pressure-assisted capillary electrophoresis-frontal analysis.

^d^ *K*_D_ = *k*_d_/*k*_a_. *k*_a_ is the binding rate constant; *k*_b_ is the dissociation rate constant.

^e^ FRET Assay means fluorescence resonance energy transfer assay.

^f^ IRa is the ratio of the relative abundance of complex ion peaks to the sum of all secondary structures and their complexes in the MS. Generally, the closer the value of IRa is to 1, the stronger the affinity of the compound for i-motif.

^g^ *K*_b_ is the binding constant.

^h^ *K*_a_ = 1/*K*_d_, which is another way to show the binding constant.

^i^ DC_50_ represents the concentration corresponding to the 50% displacement value of the compound as calculated from the fitted dose-response curve.

“-”: No related data in the corresponding literature.

“Destabilize”: The compound can cause i-motifs unfolding.

“Stabilize”: The compound makes DNA sequences folding into i-motifs or makes i-motifs more stable.

“Slight”: The interaction between the compound and i-motif is slight.

“None”: There is no interaction between the compound and i-motif.

## 2 Types of i-motifs

G4s and i-motifs transform dynamically with cell cycle progression. Consequently, there is a general consensus that both DNA structures are highly likely to participate in controlling gene expression by turning them on or off at the DNA level. Biffi et al. (2013) found that G4 structures mostly occurred during the S phase, while Zeraati et al. (2018) reported a maximum number of i-motif structures in the late G_1_ phase. Most i-motif sequences are located in the regulatory regions of the human genome (Huppert and Balasubramanian, 2005; Belmonte-Reche and Morales, 2020). Therefore, i-motifs are considered to be closely related to DNA replication, transcription, and translation of human genes, which are summarized in this section.

Promoters, just like switches, are important factors in controlling gene activity. Promoter function depends on their structure, which impacts their affinity with RNA polymerase and is associated with imbalanced gene expression and illnesses. In recent years, i-motifs located in promoters have been demonstrated to be closely connected with gene transcription. To date, many small molecule ligands have been reported, which may interact with these i-motifs and play important roles in regulating the biological activities of specific genes.

### 2.1 *Bcl-2* i-motif

*Bcl-2* is a proto-oncogene, the expression product of which is suspected as the motivator for cancers and autoimmune diseases by activating superfluous cells (Roy et al., 2014). The human *Bcl-2* gene has two main promoters, P1 and P2, both of which regulate the initiation of gene transcription (Dexheimer et al., 2006). P1 is a GC-rich promoter in which i-motif structures have been reported *in vitro* (Amato et al., 2022). Previous studies proved that G4 structures provide a negative signal, resulting in the silencing of gene expression (Brooks and Hurley, 2009). In contrast, the i-motif in the *Bcl-2* P1 promoter is a positive signal to activate gene transcription (Kang et al., 2014).

The small molecule ligand biproline amide derivative **PBP1** (**2,** Figure 2) specifically binds the *Bcl-2* i-motif, which promotes folding of the C-rich DNA sequence into i-motif structure at neutral pH and up-regulating *Bcl-2* expression in both RNA and protein synthesis. Its isomer **PBP2** (**3,** Figure 2) has a lower affinity for the *Bcl-2* i-motif and reduces the level of active caspase enzymes 3/7 in HCT-116 cells in flow cytometry analysis (Debnath et al., 2017). Yang et al. (2020) reported that the natural flavonoids **P5** and **P6** (**4, 5,** Figure 2) had a higher affinity for the *Bcl-2* i-motif compared to **P1** (**6,** Figure 2). The acridone derivative **A22** (**7,** Figure 2) (Li et al., 2020) stimulated *Bcl-2* expression, reducing hepatocyte apoptosis and alleviating inflammation, endoplasmic reticulum stress, and cirrhosis in a NAFLD/NASH (non-alcoholic fatty liver disease/non-alcoholic steatohepatitis) model. Thus, **A22** (**7**) may be a promising compound for the treatment of liver illnesses. In the quest for therapy for diffuse large B cell lymphoma (DLBCL), Kendrick et al. (2017) proposed an innovative strategy to simultaneously use GQC-05 and **IMC-76** (**8,** Figure 2), which respectively recognize the *Myc* G4 and the *Bcl-2* i-motif. This treatment not only regulated *Myc* and *Bcl-2* gene expression but also significantly decelerated tumor growth in DLBCL xenografted mice.

hnRNP LL (heterogeneous nuclear ribonucleoprotein LL) protein, which shows tissue-specific distribution, activates T cells by transferring the transcriptional genome and then advances cell proliferation and inhibits cell death (Oberdoerffer et al., 2008). Roy et al. (2016) defined hnRNP LL as an active transcription factor for *Bcl-2*, which recognizes the i-motif by binding the preferred loop to its four RRM (RNA recognition motif). Among the four RRMs, RRM1 and RRM2 strongly combine with the *Bcl-2* i-motif to drive the i-motif transformation into a more stable hairpin structure and finally upregulate *Bcl-2* transcription. Furthermore, the small molecule **IMC-48** (**9,** Figure 2) increased i-motif and hnRNP LL levels by binding the central ring of the i-motif. In contrast, **IMC-76** (**8**) wrapped flexible hairpins to decrease the levels of this compound (Cui et al., 2014; Kendrick et al., 2014).

### 2.2 *c-kit* i-motif

Located in the human chromosome at 4q12-13, the *c-kit* proto-oncogene is a key point in cancer occurrence and proliferation (Abdel-Magid, 2021). The transcriptional product of *c-kit* is a type III receptor tyrosine kinase, which participates in regulating hematopoietic stem cell proliferation and differentiation. Recent findings have shown that *c-kit* mutations are strongly related to the morbidity and prognosis of gastrointestinal stromal tumors, small-cell lung cancer, melanoma, and systemic mastocytosis (Pathania et al., 2021). Given the overexpression of *c-kit* gene in some diseases, targeting and inhibiting *c-kit* expression is considered a novel strategy.

Through spectroscopic analysis, Bucek and his team (2009; 2010) reported the presence of i-motif-forming sequences in the *c-kit* promoter, where G4s and i-motifs coexist with duplexes at pH values of 3 to 6.5. Additionally, compounds such as terpyridine derivatives (Wei and Gao, 2015), trisubstituted isoalloxazines (Bejugam et al., 2007), and benzo[a]phenoxazine derivatives (McLuckie et al., 2011) have high affinities for *c-kit* G4s.

### 2.3 *c-myb* i-motif

As a human proto-oncogene, *c-myb* plays an important role in regulating cell proliferation and differentiation in the hematopoietic and gastrointestinal systems. The *c-myb* mutation or overexpression induces cancerous lesions including acute myeloid leukemia and breast, colon, and gastroesophageal cancers (Gonda, 1998; Ramsay et al., 2003). Hence, it is vital to regulate *c-myb* expression when treating these diseases.

**FIGURE 3:**
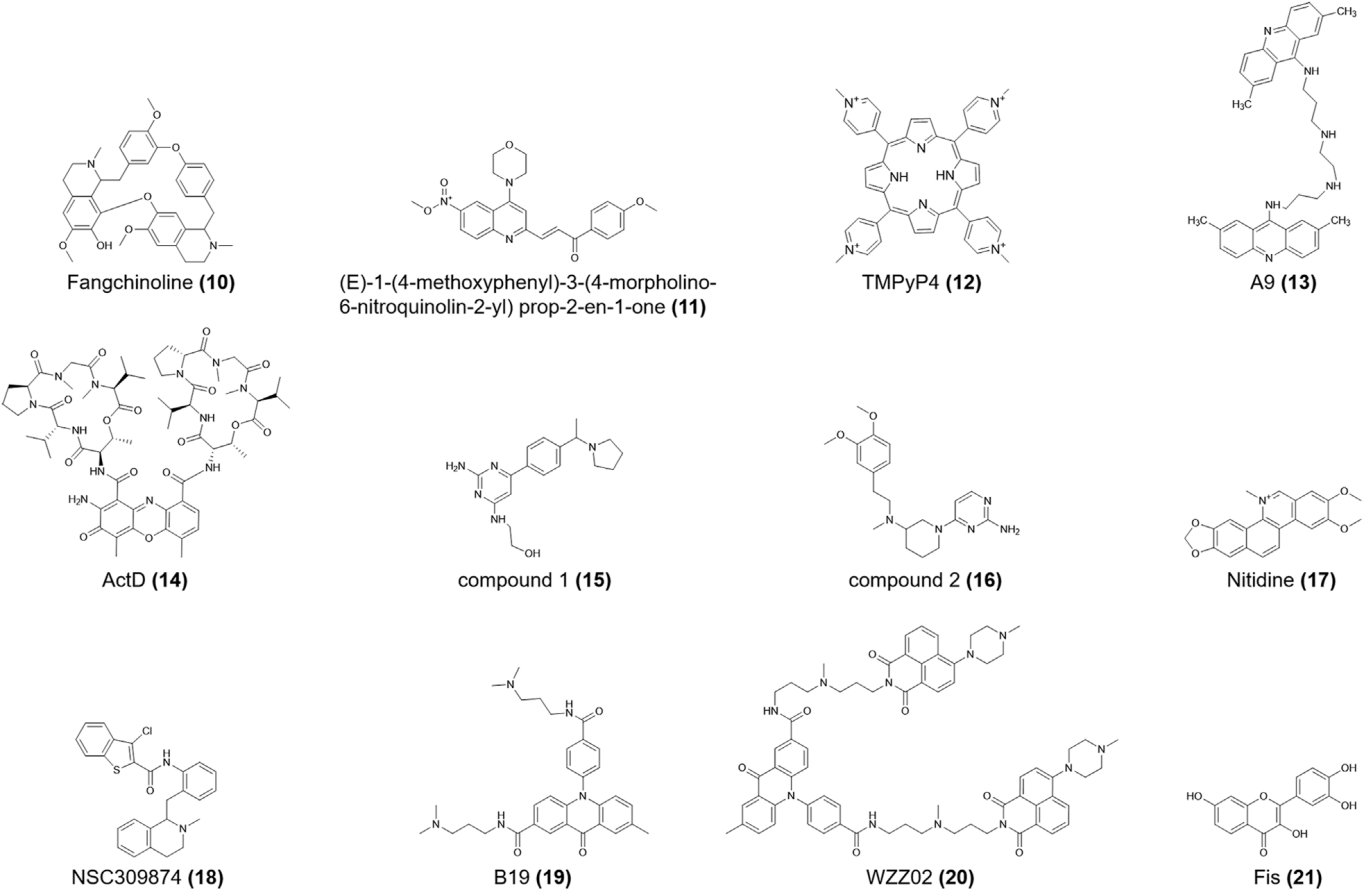
Structures of compounds (**10–21**).

The *c-myb* G4 DNA blocks the transcriptional activity of the T7 RNA polymerase (Mishra et al., 2019). Wang et al. (2020) reported that the natural compound hyoscine butylbromide specifically bound *c-myb* G4 DNA with a binding constant of 1.18 × 10^5^ L/mol. There remains comparatively scarce researches on *c-myb* i-motifs, most of which focus on factors affecting the formation and structural stability of the *c-myb* i-motif. Intramolecular i-motif structures formed in a stretch of cytosine-rich sequence S6 in the transcription start site of *c-myb* at pH 7.0 and ions like H^+^ and K^+^ promoted the transformation of the double helix to G4/i-motif structures (Li et al., 2016). Moreover, Li et al. (2016) screened out a natural product, **Fangchinoline** (**10,** Figure 3), which combines the *c-myb* i-motif DNA mainly in a “1 + 1” mode with a low affinity.

### 2.4 *c-myc* i-motif

The *c-myc* is a proto-oncogene, the protein of which widely regulates transcription (Dang et al., 2006). The *c-myc* expression has important implications for the metabolism and growth of cancers including adenomatous colorectal polyposis and lymphoma (Shim et al., 1997; He et al., 1998). Both *c-myc* G4 and i-motif structures control *c-myc* transcription, as demonstrated in footprinting analysis of the *c-myc* promoter region *in vitro*.

Heterogeneous nuclear ribonucleoprotein K (hnRNP K) is closely related to chromatin remodeling, transcription, splicing, and translation (Bomsztyk et al., 2004). For *c-myc*, it is also a significant transcriptional activator that may speed carcinogenesis (Sutherland et al., 2016) **(E)-1-(4-methoxyphenyl)-3-(4-morpholino-6-nitroquinolin-2-yl) prop-2-en-1-one** (**11,** Figure 3) is the first-reported ligand for the hnRNP K protein. When this compound binds to hnRNP K, it induces i-motifs unfolding, resulting in *c-myc* downregulation. It also shows different anti-proliferative effects on human cancer cell lines, with IC_50_ values ranging from 1.36 M to 3.59 M (Shu et al., 2019). **TMPyP4** (**12,** Figure 3) specifically binds to the top of the i-motif structure of nuclease hypersensitivity element III1 (NHE III1) in *c-myc*, subsequently preventing hnRNP K from binding to *c-myc* and ultimately silencing *c-myc* (Bialis et al., 2007). According to ITC data, **TMPyP4** (**12**) can embed itself in two adjacent C-C^+^ base pairs through π–π stacking (Figure 7). Qin et al. (2017) confirmed that **TMPyP4** (**12**) can bind to the major external groove of the i-motif structure owing to its large steric hindrance and ionic strength (Figure 7). **TMPyP4** (**12**) also combines with both ends of the i-motif in the presence of Van der Waals and electrostatic forces (Cashman et al., 2008) (Figure 7).

The double acridine derivative **a9** (**13,** Figure 3), which stabilizes the *c-myc* promoter NHE III1 G4 and i-motif, inhibited the proliferation of lymphatic carcinoma cell lines, including Raji and CA46, and limited the clone formation and migration of the SiHa human cervical squamous cell line. And the IC_50_ of these two lymphatic carcinoma cell lines are 3.385 μM and more than 50 μM respectively (Kuang et al., 2020). **ActD** (**Actinomycin D**) (**14,** Figure 3) binds non-specifically to the duplex DNA and unwinds it, after which **ActD** (**14**) preferentially binds to G4 DNA by terminal stacking, with a binding constant of 1.34 × 10^5^ M^-1^ at pH 7.2. However, the binding constant of **ActD** (**14**) and *c-myc* i-motif is only 9.3 × 10^4^ M^-1^ at pH 5, which is the weakest constant among three non-B DNA conformations (Niknezhad et al., 2016).

### 2.5 *RAS* i-motif

The *RAS* gene family is one of the most widespread proto-oncogenes and includes *KRAS*, *NRAS*, and *HRAS*. HRAS transmits signals to the nucleus and stimulates cell proliferation (Lowy, 1993). Miglietta et al. (2015) proved that the lateral loops of the *HRAS* i-motif provide binding sites for the RRM domains of heterogeneous nuclear ribonucleoprotein A1 (hnRNP A1). After hnRNP A1 stably binds to the i-motif sequence, gene transcription is activated. **Compounds 1** and **2** (**15, 16,** Figure 3) show sub-micromolar affinities for the *HRAS* i-motif (Table 1), which is a C-rich sequence located upstream of the *HRAS* oncogene transcription start site. **Compound 1** (**15**) is assumed to directly bind to the i-motif core region, while **compound 2** (**16**) binds to the minor loop region (Journey et al., 2018).

Among the *RAS* gene family, *KRAS* is a frequently mutated oncogene with specific frequencies of variation in different cancer types. *KRAS* hyperactivation often results in sustained tumor proliferation, mostly common in lung, colorectal, and pancreatic carcinomas (Friday and Adjei, 2005; Baines et al., 2011; Cox et al., 2014). Thus, researchers have identified small molecule inhibitors with anticancer activity to treat *KRAS*-driven cancers (Kumar and Priya Doss, 2021a; Kumar and Priya Doss, 2021b; Udhaya Kumar et al., 2022).

While studies on the *KRAS* i-motif are insufficient, it is clear that the C-rich nucleic acid sequences in the *KRAS* promoter can form i-motifs, which are in a state of dynamic transition with hairpin species (Manzini et al., 1994; Amato et al., 2022). HnRNP K can bind selectively to the *KRAS* i-motif and upregulate *KRAS* transcription. Kaiser et al. (2017) showed that **nitidine** (**17,** Figure 3), a benzophilite alkaloid, is preferred to bind hairpin species in the central loop region of *KRAS* i-motifs (Figure 7). The cross-link between hnRNP K and i-motif is then destroyed, following with refolding of the i-motif and increasing gene transcription. **Nitidine** (**17**) also stabilized *KRAS* G4s and microscale thermophoresis analysis showed that it decreased *KRAS* expression in pancreatic cancer cells (Morgan et al., 2016). Thus, **nitidine** (**17**) plays a dual role in i-motif and G4s, providing a distinct mechanism for drug development.

### 2.6 *PDGFR* i-motif

Brown et al. (2017) reported that the nuclease hypersensitivity element (NHE) in the human *PDGFR* promoter could form an i-motif structure. The R1 mutation (T-to-C) in the *PDGFR* i-motif, increased the thermal stability of the i-motif (Δ*T* = 13.2 °C). **NSC309874** (**18,** Figure 3) preferentially bound to the R1-mutated i-motif in medium-flux screening and finally downregulated gene expression in neuroblastoma cells.

The acridone derivative **B19** (**19,** Figure 3) selectively induced i-motif formation at the *c-myc* promoter and downregulated its transcription, eventually causing apoptosis in cancer cells (Shu et al., 2018). Since the compound showed insufficient anticancer activity, Zhang et al. (2021) modified it into the acridone naphthalide derivative **WZZ02** (**20,** Figure 3). *In vitro* experiments showed that **WZZ02** (**20**) exhibited the specificity of stabilizing the G4 located in the *PDGFR* promoter and the potential to disrupt the complementary i-motif structure, leading to *PDGFR* downregulation in a dose-dependent manner. **WZZ02** (**20**) also showed excellent anticancer activity in the MCF-7 xenograft tumor model by inducing cell apoptosis and cycle arrest and inhibiting proliferation, likely due to its intricate interaction with *PDGFR* G4 and i-motif. Hence, **WZZ02** (**20**) may be a potential agent for cancer therapy.

### 2.7 *RAD17* i-motif

The cell cycle checkpoint protein RAD17 is directly involved in the cellular DNA damage and replication detection system, playing a critical role in maintaining genomic stability and carcinogenesis (Zhou et al., 2013). While limited work has assessed i-motifs located in the *RAD17* promoter sequence, the structure of the *RAD17* i-motif changes rapidly when the pH drops, without observable hysteresis (Rogers et al., 2018).

### 2.8 *RET* i-motif

The *RET* proto-oncogene encodes the receptor tyrosine kinase and is crucial for neurodevelopment. Most commonly, *RET* mutations cause two neural crest disorders, Hirschsprung’s disease and multiple endocrine neoplasia type 2 (Manié et al., 2001). Stable G4 and i-motif structures can form within the subterminal promoter region of human *RET* (Huppert and Balasubramanian, 2007). A fluorescent cytosine analog, 1,3-diaza-2-oxophenothiazine used as a pH probe showed DNA forming double strands at higher pH while shaping i-motifs in acidic environments. The *RET* i-motif also showed a higher anisotropic signal; thus, this system can be used to monitor reversible pH changes in the design of molecular logic gates and intricate biosensors (Bielecka and Juskowiak, 2015; Bielecka et al., 2019).

### 2.9 *VEGF* i-motif

Vascular endothelial growth factor (VEGF) is a diffusible endothelial cell-specific mitogen and angiogenic factor and the major regulator of physiological angiogenesis irreplaceable for embryonic development and disease progression. Additionally, *VEGF* mRNA upregulation is associated with angiogenesis in most common human tumors and proliferative retinopathy. Thus, resisting *VEFG* is considered an effective strategy for the treatment of neoplasms and retinopathy (Ferrara, 1995; Matsumoto and Ema, 2014).

Guo et al. (2008) reported the presence of intramolecular i-motif structures in the poly C region of the proximal end of the *VEGF* promoter. In the context of epigenetics, accompanied by the cytosine-phosphate-guanine (CpG) methylation, *VEGF* i-motif structures could be more stable (Kimura et al., 2022). With the help of a DNA methylation detection system, Yoshida et al. (2016) showed decreased DNA polymerase efficiency with increased DNA methylation in *VEGF* i-motif sequences, indicating that the i-motif formation sequence may inhibit gene expression by increasing methylation. Takahashi et al. (2020) showed that the plant flavonol **fisetin** (**Fis**) (**21,** Figure 3) preferred to combine with the *VEGF* i-motif and provoked i-motif unfolding. Moreover, the fluorescence emission spectra showed that **Fis** (**21**) bound to the central ring of the *VEGF* i-motif.

### 2.10 Telomere i-motif

Telomerase maintains the integrity of chromosome ends and is important in cell immortalization and carcinogenesis. Human telomerase mainly consists of three different subunits, among which the catalytic subunit hTERT is the rate-limiting determinant of telomerase activity. The hTERT expression is sensitized when carcinogenesis occurs. However, this expression is regulated not only by various activators and inhibitors but also epigenetic pathways like DNA methylation and histone modification (Takakura et al., 1999; Daniel et al., 2012).

**FIGURE 4:**
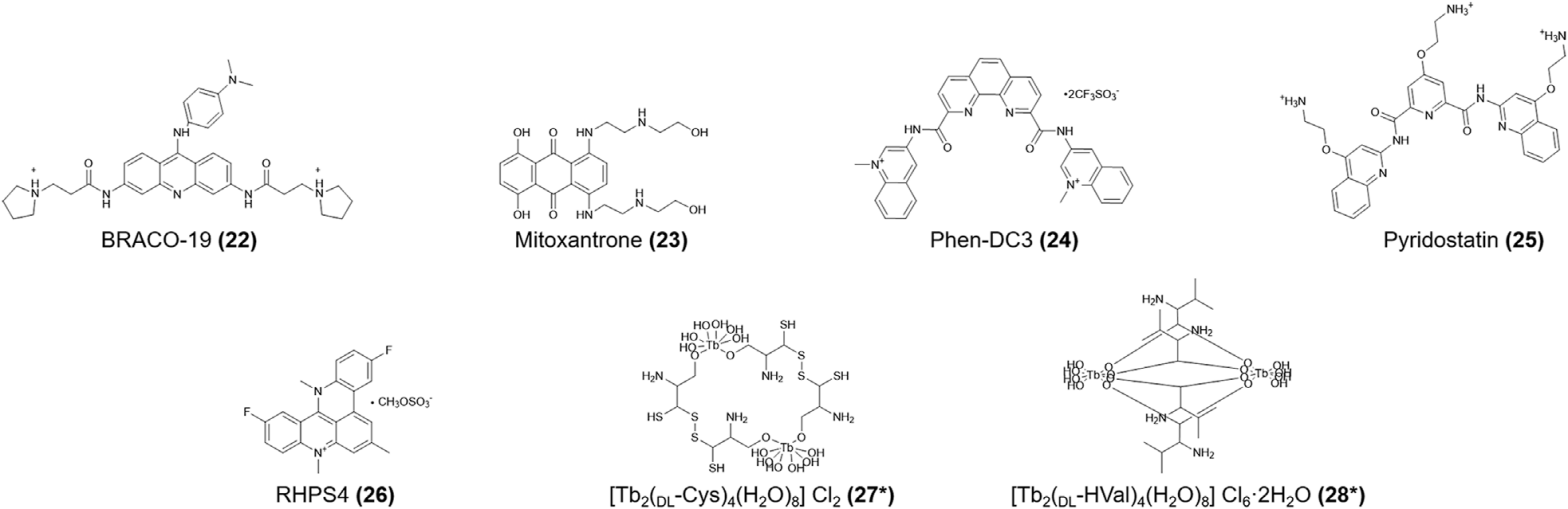
Structures of compounds (**22–28**). *Lattice water and chloride ions are omitted.

Initially, **TMPyP4 (12**) was identified as a specific ligand for hTERT G4s (Fedoroff et al., 2000). Later, NMR spectroscopy results from Fernández et al. (2011) showed that **TMPyP4** (**12**) also advances the formation of the h-telo (human telomeric) i-motif DNA structure in a non-intercalation mode. Pagano and colleagues (2018) showed that existing G4 ligands, including **BRACO-19**, **mitoxantrone**, **phen-DC3**, **pyridostatin,** and **RHPS4** (**22-26,** Figure 4) also interact with the h-telo i-motif. Moreover, **BRACO-19** (**22**), **mitoxantrone** (**23**), and **phen-DC3** (**24**) destabilize the h-telo i-motif. However, Wright and his team (2016) reported the opposite finding, likely due to the different experimental conditions, in which **mitoxantrone** (**23**) firmly bound to the i-motif and induced its formation at pH 5.5. As **mitoxantrone** (**23**) is a well-known topoisomerase II inhibitor for the treatment of non-Hodgkin lymphoma and metastatic breast cancer and slowing the progression of multiple sclerosis, the mechanisms by which **mitoxantrone** (**23**) combines with the i-motif require urgent study.

**FIGURE 5:**
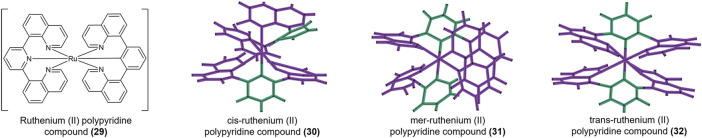
Structure of **[Ru**(**bpp)**_**2**_ **]** ^**2+**^ (**29**). Crystal structures of **cis** (**30**), **mer** (**31**), and **trans** (**32**).

**[Tb**_**2**_(_**DL**_ **-Cys)**_**4**_ **(H**_**2**_ **O)**_**8**_ **]Cl**_**2**_ and **[Tb**_**2**_(_**DL**_ **-HVal)**_**4**_ **(H**_**2**_ **O)**_**8**_ **]Cl**_**6**_ **.2H**_**2**_ **O** (**27, 28,** Figure 4) are terbium amino acid complexes that can bind not only h-telo G4s but also i-motifs without conformational changes (Xu et al., 2006). The models for the interactions between the h-telo i-motif and a **ruthenium (II) polypyridine compound** (**29,** Figure 5) are complicated. Especially, the **cis** isoform (**30,** Figure 5) can bind the major groove of the h-telo i-motif more tightly due to its smaller spatial structure compared to the **mer** (**31,** Figure 5) and **trans** (**32,** Figure 5) isoforms. Three isoforms combine with the i-motif in different ways: the Λ-cis has 68 contacts with the i-motif core, only 52 in Δ-cis, and even fewer in the **mer** (**31**) and **trans** (**32**) isoforms. In addition, luminescence lifetime data supported that the **cis** (**30**) isoform can be used as a small molecule to detect death-associated protein (DAP) i-motifs (Spence et al., 2020).

**FIGURE 6:**
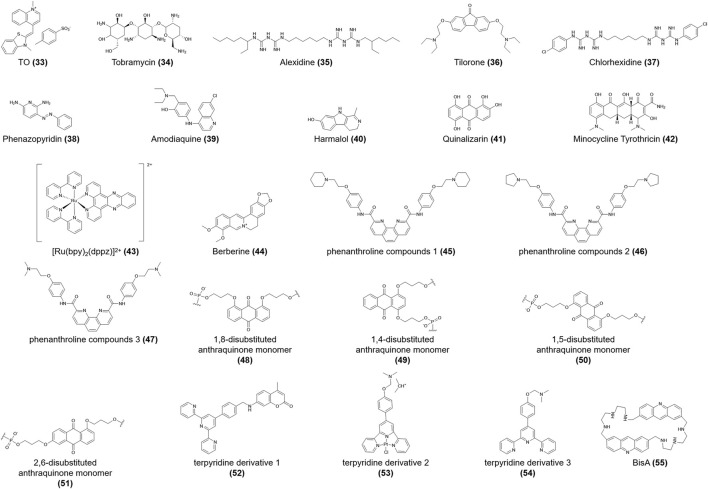
Structures of compounds (**33–55**).

Sheng et al. (2017) reported that **thiazole orange** (**TO**) (**33,** Figure 6) stabilized the h-telo i-motif no matter the sequence, therefore available as probes in i-motif DNA analysis. Moreover, they identified several novel i-motif-binding ligands, including **tobramycin**, **alexidine**, **tilorone**, **chlorhexidine**, **phenazopyridine**, **amodiaquine**, **harmalol**, **quinalizarin,** and **minocycline tyrothricin** (**34-42,** Figure 6). Recent research is lacking regarding how these compounds interact with i-motifs; thus, further exploration is needed. Slightly interacting with i-motif DNA, **[Ru**(**bpy)**_**2**_ **(dppz)]** ^**2+**^ (**43,** Figure 6) preferentially binds the h-telo G4 sequence and can act as a “photoswitch” to monitor the dynamic transition of G4 DNA structures (Shi et al., 2010).

**Berberine** (**44,** Figure 6) binds to the h-telo i-motif structure *via* electrostatic interactions, with a weak dissociation constant (Table 1) (Xu et al., 2016; Gargallo et al., 2021). **Berberine** (**44**) can also be used as an effective fluorescent probe to monitor the conformational conversion of i-motifs driven by pH (Xu et al., 2016). Absorption titration and thermodynamic analyses showed that **phenanthroline compounds 1–3** (**45-47,** Figure 6) bind to the C-C^+^ base pairs of telomeric i-motifs in the presence of π-π stacking, intercalation, or hydrophobic interaction. These compounds show a higher affinity for G4 DNA compared to the i-motif, which is attributed to telomeric G4s having larger π-π stacking interactions and van der Waals contacts (Wang et al., 2013). The **1,8-, 1,4-, 1,5-, and 2,6-disubstituted anthraquinone monomers** (**48-51,** Figure 6) were investigated to assess their effects on the thermal stability of the i-motif. When anthraquinones modify the TAA loop of the h-telo DNA sequence in the intercalation or external binding mode, the melting temperature increases significantly, accordingly enhancing the stability of the i-motif structure (Gouda et al., 2017).

In their analysis of structure-activity relationships, Wei et al. (2015) reported that the coumarin group may be the determinant of the specific affinities of **terpyridine derivatives 1–3** (**52-54,** Figure 6) for the h-telo i-motif, among which **terpyridine derivative 1** (**52**) reduced 88.7% of the telomerase activity and slightly restrained the bioactivity of topoisomerase I activity at 5 μmol/L. Moreover, **macrocyclic bis-acridine** (**BisA**) (**55,** Figure 6) shows a considerable affinity for the telomeric i-motif. While **BisA** (**55**) did not show obvious effects on the physiological function and conformation of telomeric i-motifs (Bonnet et al., 2022).

In addition, chemically modified cytosines have different effects on the formation of i-motif structures under physiological conditions. The telomeric i-motif became less stable at a neutral pH due to the methylated cytosine in MCF7 and MCF10A cell lines (Wright et al., 2020). Xu et al. (2015) also reported that the telomeric i-motif was stable with small amounts of cytosine methylation modification. However, numerous hydroxymethylation and methylation modifications could lead to structural destabilization. In plants, cytosine methylation is similarly meaningful in the epigenetic regulation of telomeric DNA (Školáková et al., 2020), which can also fine-tune the stability and the pH dependence of i-motifs. Balasubramaniyam et al. (2021) reported that the halogenation of cytosine at C5 accelerated i-motif folding, which eventually altered the pH dependence. Moreover, the hydrophilic hydroxyl group at C5 is more tolerant to immunostimulation compared to the hydrophobic methyl group (Kandimalla et al., 2001). Therefore, modifying the deoxycytidine or cytidine of i-motifs may be a new strategy to regulate immunity *in vivo*.

### 2.11 Other i-motifs

**FIGURE 7:**
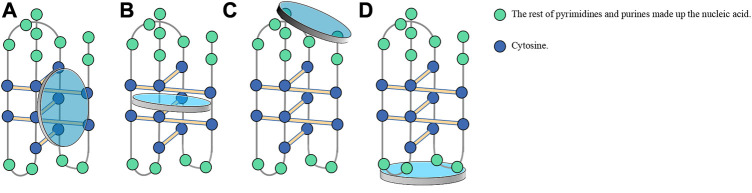
Usual modes of ligands binding to the i-motif. **(A)** Binding to the major external groove of the i-motif owing to large steric hindrance and ionic strength. **(B)** Embedding in C-C^+^ base pairs by π-π stacking. **(C)** Stacking at two ends of the i-motif in the presence of Van der Waals and electrostatic forces. **(D)** Binding to hairpin species in the central loop region of the i-motif due to steric hindrance and molecular length.

The synthetic C6T i-motif has two unequal broad grooves and is used to explore possible binding modes with compounds with long molecular structures such as polyamines. **Putrescine** (**56,** Figure 9) with the shortest molecular length, only binds to the loop region (Figure 7) and, thus, has the lowest affinity for i-motifs. In contrast, the longer molecule **spermidine** (**57,** Figure 9) can bind the loop region and the groove; therefore, it has two quite different binding constants. The longest molecule, **spermine** (**58,** Figure 9), can simultaneously bind two broad grooves of the C6T i-motif, with the highest binding affinity (Molnar et al., 2019) (Table 1). Based on mechanisms of polyamine binding to i-motifs, additional research is needed to identify additional ligands with greater affinity by altering the molecular length of the polyamines. Kinetic analysis has shown that the addition of 5′-terminal guanidino-i-clamp (Tsvetkov et al., 2019) or i-clamp (Tsvetkov et al., 2018) can decrease the unfolding rate of synthetic i-motifs, which guarantees the stability of i-motifs. It is also possible to become unstable given the steric hindrance.

Additionally, BmPOUM2 has been reported to be a significant regulator of the wing disc cuticle protein gene, namely, BmWCP4. Silkworms cannot complete metamorphosis when the BmPOUM2 is expressed. An i-motif structure and the novel BmILF i-motif-binding protein have been identified. BmILF specifically binds the i-motif and activates BmPOUM2, which prevents silkworm metamorphosis into moths (Niu et al., 2018). For agriculturists, these findings provide new insights into the epigenetic mechanism for how to control the growth of silkworms to achieve a high silk yield.

The above summary showed that natural i-motif structures are mainly located in the promoter regions of various oncogenes. Hence, most current studies on the biological function of i-motifs have focused on how to build targeted and effective anticancer strategies through the combination of i-motif and other molecules. Therefore, attention to genes with very few or no drug studies, such as *c-kit*, *c-myb*, *RAD17*, and *RET*, may demonstrate the value and application of i-motifs in the medical field. We hold a strong belief that i-motifs in promoters can be subtly regulated by ligands under specific conditions to stabilize or disrupt their structures and, ultimately, regulate their biological functions.

## 3 Diseases closely associated with i-motifs

The discussion above provides a preliminary conclusion that i-motif-forming sequences are crucial for regulating chromosome integrity, mitosis, and protein synthesis and are linked to challenges such as cancer and aging (Hampton, 2011; Armanios and Blackburn, 2012; von Figura et al., 2009). The following section discusses in detail diseases related to i-motifs.

### 3.1 Diabetes mellitus (DM)

DM is often accompanied by cardiovascular complications like coronary heart disease, which is a common reason for clinical death (Rogowicz-Frontczak et al., 2012). Therefore, it is particularly urgent to treat diabetes. The insulin minisatellite region is the insulin-linked polymorphic region (ILPR), the polymorphism length of which is strongly linked to the genetic susceptibility of insulin-dependent diabetes mellitus (IDDM) (Catasti et al., 1996). The C-rich duplex sequence in ILPR forms intermolecular/intramolecular i-motifs through pairing between C^+^ and C (Jolad et al., 2005; Dhakal et al., 2012). Catasti et al. (1997) suggested that the stable folding of the i-motif in C-rich sequences led to the loose structure upstream of the insulin gene, promoting insulin expression. Dhakal et al. (2010) indicated that, simply from a mechanical perspective, when insulin i-motifs interact with RNA polymerases, the unfolding force of the i-motif is greater than the stall force, interrupting gene transcription (Galburt et al., 2007).

Experimental results at the cellular and individual levels will reveal whether insulin i-motifs advance or inhibit insulin transcription and provide more therapeutic options for patients with DM. Thus, more studies are needed on i-motifs in the human insulin gene at these levels.

### 3.2 HIV

HIV-1 is a human immunodeficiency virus and a basic pathogen of acquired immunodeficiency syndrome (AIDS) (Radestock et al., 2013). The i-motif structure can also form in the promoter of the HIV-1 DNA genome. Moreover, the i-motif located in the long terminal repeat (LTR) of the HIV-1 promoter has a unique folding pattern that differs from that in the human genome. Ruggiero et al. (2019) reported that hnRNP K induces the formation of the HIV-1 LTR i-motif and subsequently represses the transcription, finally decreasing the virulence of HIV-1. These findings lay a theoretical foundation for innovative antiviral drug design based on the selective recognition of the HIV-1 i-motif.

### 3.3 Neuropsychiatric disorders

Depression is a neuropsychiatric disorder strongly associated with the serotonin transporter (SERT). The linkage polymorphic region of SERT contains two C-rich allelic variants that regulate susceptibility to depression by altering SERT expression levels. Both variants can form i-motifs; however, the mechanism requires further exploration (Zhang et al., 2015; Thorne et al., 2021). As a key regulator of serotonin, i-motifs in the SERT-linkage polymorphic region will be an important pharmacological breakthrough for treating depression.

Fragile X syndrome (FXS) is the most common consequence of genetic intellectual disability caused by the CGG/CCG tandem repeat sequences of Fragile X Messenger Ribonucleoprotein 1 (FMR1) on the X chromosome. CCG repeats prefer to form stable intermolecular i-motif structures (Tekendo-Ngongang et al., 2021; Zhang et al., 2022). Chen et al. (2018) reported that when the CCG trinucleotide repeat region is bound to the **Co^II^ (Chro)**_**2**_ dimer (**59,** Figure 9), the i-motif unfolds and is restored the double-helix structure, suggesting that **Co^II^ (Chro)**_**2**_ (**59**) could be a new drug for the treatment or diagnosis of neurological diseases.

In addition, hexanucleotide repeat amplification sequence G4C2 in the C9orf72 gene is the most common single genetic factor for frontotemporal dementia and amyotrophic lateral sclerosis. G4C2 can fold into unusual secondary structures, including R-loops, i-motifs, and G4s (Kumar et al., 2016). These findings indicate that i-motifs can be a new target in gene therapy for neuropsychiatric disorders.

### 3.4 Cancer

Tumor occurrence is often accompanied by gene mutations. Any gene abnormality, such as the inactivation of the anti-oncogene or the activation of the proto-oncogene can cause cancer. Recent reports have demonstrated the central role of *Bcl-2* in orchestrating the interplay between apoptosis and senescence. *Bcl-2* over-activation results in the abnormal proliferation of cancer cells (Nahta and Esteva, 2003; Kim et al., 2004). As one of the most important transcription factors, c-myc protein is particularly significant for the reprogramming of multiple types of cancer cells, as well as their proliferation and chemoresistance (Fatma et al., 2022). The *c-myc* inactivation can lead to sustained tumor regression, which may be a key therapy to reverse cancerous growth and restore antitumor immune responses in patients with high *c-myc* expression (Dhanasekaran et al., 2022). Additionally, telomeres and *VEGF* play vital roles in indefinite proliferation and nutrient supply, which are essential for tumor growth.

As i-motifs form in specific regions of most oncogenes, a link must exist between cancer development and therapy. As mentioned above, researchers often establish specialized models, screen and modify drugs with certain cytotoxicity, and use the effects between drugs and i-motif to verify the anticancer potential of the studied drugs. For example, in the anti-DLBCL model, **IMC-76** (**8**) was found to bind the *Bcl-2* i-motif and upregulate *Bcl-2* expression, thus slowing cancer cell growth (Kendrick et al., 2017). In the anti-lymphoid Raji cell line model, **a9** (**13**) interacted with the *c-myc* i-motif and showed high cytotoxic effects, with an IC_50_ reaching 3.385 μM (Kuang et al., 2020). In the cancer model associated with *VEGF*, **Fis** (**21**) was used as a probe to specifically recognize the *VEGF* i-motif; therefore, this system can be used to diagnose *VEGF*-associated cancers (Takahashi et al., 2020). In the cancer model associated with telomeres, **terpyridine derivative 1** (**52**) binds to the h-telo i-motif to inhibit telomerase and topoisomerase activity (Wei and Gao, 2015). Table 1 summarizes studies on drugs. Although clinical studies on these potential anti-cancer drugs are lacking, those on the association of i-motifs with cancer are essential.

Since these oncogenes have been introduced above, the retinoblastoma gene *Rb* is highlighted here. *Rb* is the first tumor suppressor gene identified in humans, the functional incapacitation of which is related to retinoblastoma tumorigenesis. The product of *Rb*, pRB protein, controls the cell cycle transition from the G_1_ to the S phase, pausing the cell cycle in a static state. Clinical findings suggest that a dysregulation of the G_1_-S control pathway may occur in retinoblastoma and sporadic lung, breast, and bladder cancers (Dannenberg et al., 2000). Thus, restoring or enhancing *Rb* function may be a late-model strategy for these cancers. Lee et al. (2015) identified the *Rb* i-motif based on the pH, providing evidence for retinoblastoma diagnosis. Studies on the structural characteristics, biological functions, and targeted ligands of i-motifs in *RB* are scarce (Xu and Sugiyama, 2005) and require future exploration.

In addition to these diseases, i-motifs also have specific roles in fighting bacterial infections. Hemiprotonic phenanthroline-phenanthroline^+^ compounds are synthesized based on the unique structure of half-protonated nucleotide base pairs of the i-motif DNA from oncogenes. These compounds not only selectively resist tumors but also have broad-spectrum antibacterial activity, providing new therapeutic candidate drugs for patients with cancer accompanied by infection (Zhao et al., 2022).

**FIGURE 8:**
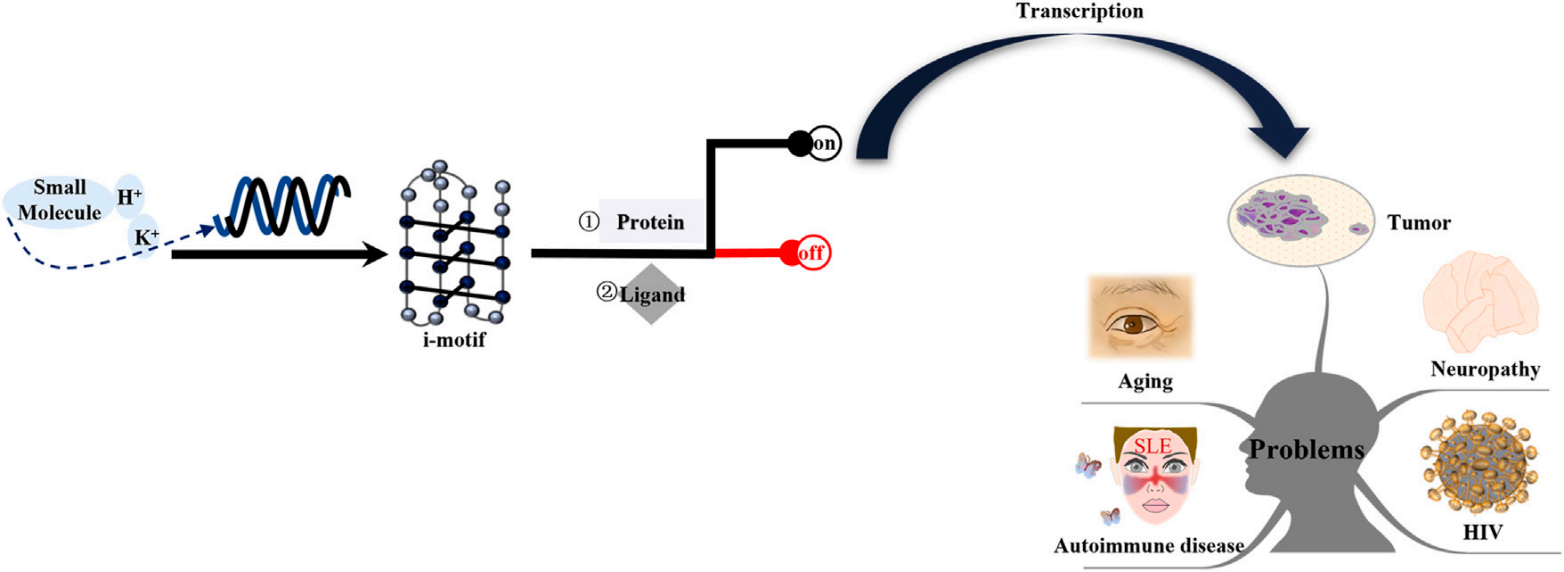
Mechanism of i-motifs *in vivo*. The i-motif DNA sequence folds into i-motif structures in specific conditions, including acid pH, K^+^, or some small molecules. I-motifs mainly have two effects: firstly, they directly bind to proteins such as hnRNP A1, hnRNP K, and hnRNP LL; second, they can directly interact with ligands. The result is switching gene expression on or off. Activated genes produce corresponding proteins after transcription. These proteins interact with other factors and form complexes that are transported to certain tissue. The release of these proteins can lead to tumors, neuropathy, aging, HIV, and autoimmune disease.

The diseases discussed in this review are i-motif-related. Among these diseases, cancer is a primary focus. The study of these diseases should consider the following four aspects: the specific gene locus of the i-motif, the effect of i-motif formation on the disease, pathways involved in the i-motif regulation of the disease, and different drug design schemes for different signaling molecules (Figure 8). As described above, current research on these diseases is still lacking. Therefore, additional research on this topic is needed.

## 4 Applications in precision medicine

Applications of i-motifs have been reported in different fields. This section introduces and describes the applications of i-motifs in medicine.

In recent years, gene-based medicines wrapped in microparticles have been successfully delivered into the human body to concentrate in the reticuloendothelial system *in vivo*, thus realizing the targeted transportation of medicine. As a drug delivery system (DDS), the nanomaterial-i-motif DNA DDS has been widely applied due to its excellent biosafety and simplicity of synthesis.

Aptamers are usually short oligonucleotide sequences or polypeptides that can bind the corresponding ligands with high affinity and strong specificity and have become valuable molecular tools in the development of bioanalysis and targeted therapy. A structure-switchable aptamer (SW-Apt) with an i-motif is one reported tool. The i-motif forms under an acidic pH, thus making an SW-Apt with high binding capacity to target cells, which modulates the specific recognition of the aptamer with the help of the dependence of the i-motif on pH and is enlightening for aptamer construction (Li et al., 2018). The aptamer DNA-cyclodextrin makes the system freely slide along the polymer PEG chain and the assembly of drugs and the i-motif DNA can respond to pH change to release drugs under intracellular acidic conditions, enhancing cellular uptake and healing efficacy (Jang et al., 2017). A DNA nanocapsule with an acidic pH-responsive i-motif DNA and a tumor cell-specific aptamer is first triggered by an acidic pH and then targets cells *via* the specific aptamer recognition and releases the carried drug doxorubicin, successfully providing selective cytotoxicity to cancer cells (Yuan et al., 2022). Similarly, a nanocarrier based on bovine serum albumin and DNA including a pH-responsive i-motif and a cancer cell-targeted guanine-quadruplex-structured aptamer also showed accurate targeting and efficient therapeutic effect on cancer cells (Yu et al., 2021). In addition to targeted therapy, i-motifs are also useful in immunotherapy. As cyclic dinucleotides (CDNs) like c-di-GMP (CDG) are agonists for stimulator of interferon genes (STING), STING-activating DNA nanovaccines (STING-NVs) with i-motif DNA on the surface showed potential to improve immunosuppression *in vitro* and *in vivo* in a murine melanoma model. In acidic environments such as the cell endosome, the i-motif was formed and CDG was released, facilitating the cell delivery of CDG (Zhang et al., 2020). Nanotubes of the anti-inflammatory drug dexamethasone modified with the i-motif were used to target the reticuloendothelial system. The i-motifs manipulated the intracellular release of dexamethasone with pH changes, thereby regulating the anti-inflammatory activity of macrophages (Sellner et al., 2017).

Hydrogels equipped with functional nucleic acids can be used to construct molecules based on aptamers, DNA enzymes, i-motif nanostructures, siRNA, and CpG oligonucleotides, all of which provide additional recognition sites, catalytic activities, and therapeutic potential (Li et al., 2016). DNA nanogels containing gemcitabine enhance the anticancer activity, for the intramolecular i-motif structure forming under acidic conditions, which facilitates nanogel disintegration and gemcitabine release (Pan et al., 2019). An intelligent DNA nanosystem based on controllable DNA nanohydrogels consists of a pH-responsive i-motif sequence, doxorubicin, the CpG fusion sequence, and an aptamer for immunostimulation and chemotherapy. This nanosystem integrates targeting, immune response, and chemotherapy to fight malignancy (Wei et al., 2019).

**FIGURE 9:**
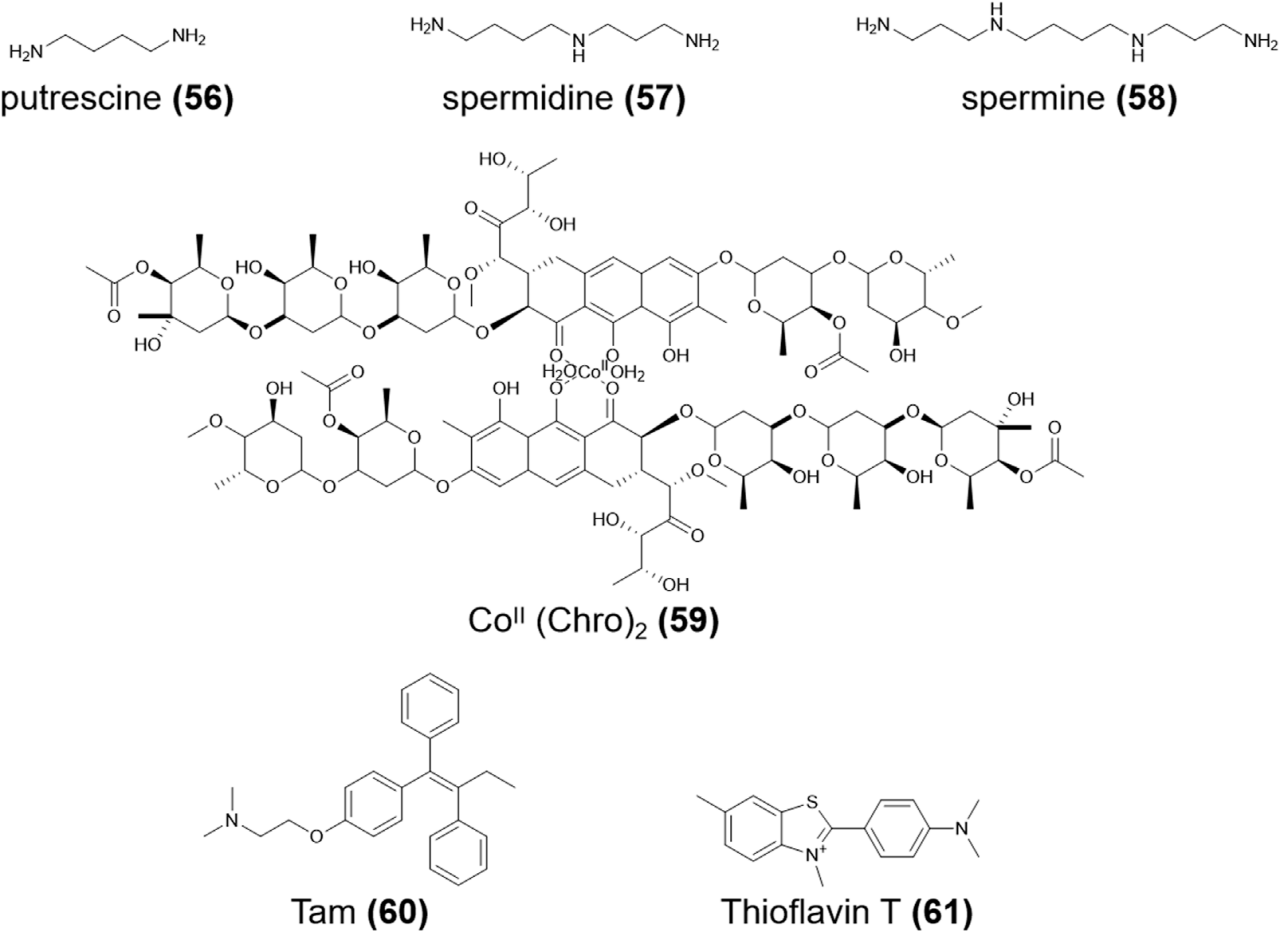
Structures of compounds (**56–61**).

In addition to triggering drug release under certain conditions, i-motifs can also act as diagnostic biomarkers in biomedical sciences. Heydari et al. (2016) used **tamoxifen** (**Tam**) (**60,** Figure 9) as the ligand and prepared an electrochemical biosensor with a nanosilica-modified carbon paste electrode to detect and differentiate human telomeric DNA. In acidic environments, the interaction between i-motifs and **Tam** (**60**) produced oxidation peaks; when the pH goes increased, the interaction decreased and the oxidation peak changed. Takahashi et al. (2020) reported the excited-state intramolecular proton transfer reaction was significantly induced and the intensity of the tautomer emission band of **Fis** (**21**) was enhanced when **Fis** (**21**) bound to the *VEGF* i-motif, which could be used for the diagnosis of cancers associated with *VEGF*. In addition, **thioflavin T** (**61,** Figure 9) can distinguish structural changes in *RET* and *Rb* i-motif sequences based on pH changes; thus, it can be used as a special probe for these two nucleotide sequences and may play a good supporting role in genetic diagnosis and treatment (Lee et al., 2015). A metal-organic framework (MOF)-shell-confined i-motif-based pH probe strategy can be used to distinguish metabolic behaviors of cancer and normal cells based on the pH, in which the labeled i-motif optimizes for pH sensing and the MOF shell limits endocrine acid diffusion (Yang et al., 2021). The dual-pyrene-functionalized i-motif can provide analytical results regarding pH changes; thus, it shows potential as a pH-sensitive fluorescent probe (Dembska et al., 2013). Likewise, i-motifs tethered on framework nucleic acids can act as a controlling unit and can be designed as logical sensors in response to extracellular changes in H^+^ and K^+^ (Peng et al., 2020).

Regarding the application of i-motifs, we mainly introduce those in nanomedicine. These studies demonstrated their important roles as detectors and in DDS made of i-motifs and nanomaterials. The application of i-motifs in the medical field is promising and warrants further exploration. In addition to the medical field, other practical applications of i-motifs are possible; however, the specific direction requires further exploration.

## 5 Conclusion

This review clarified the structure, ligands, related diseases, and applications of various i-motifs. As an emerging cytosine-rich non-B-DNA structure, i-motifs form in the promoters of many human oncogenes. As discussed above, i-motifs have significant biological functions. To elucidate the concrete regulation mechanisms of i-motifs, we extensively discussed the relevant ligand compounds. The analysis and summarization of previous research results showed that the same ligand compound can show different results for different experimental conditions (Table 1). The effects of ligands on the i-motif also vary among genes. Increasing numbers of ligands have been reported. Table 1 summarizes ligand compounds that interact with i-motifs under different experimental conditions. These ligands affect the stability of i-motifs, activate/inhibit the gene expression in many promoters, and have important implications for cell growth and development, which are closely related to cancers, mental disorders, aging, infection, and other diseases.

G4 DNA binds its ligands mainly through three modes; namely, end accumulation, intermolecular insertion, and non-specific binding; for example, porphyrinoids, telomestatin, and Se2SAP (Rezler et al., 2005; Baker et al., 2006). Figure 7 shows the major modes by which ligands bind to i-motifs. However, continuing research will reveal novel ligands and new modes. Therefore, this topic warrants additional research to explore i-motif ligand drugs, explain the regulatory mechanisms at micro and macro levels, and provide more new drugs and treatment methods for genetically-related human diseases.

Regarding cancer treatment, previous studies hypothesized that the inhibition of i-motif formation or decreasing the structural stability to fight cancer. Researchers often target a regulatory factor, which can be an enzyme, protein, or receptor molecule. Interactions between i-motifs and ligand molecules change the function of regulatory factors to downregulate or upregulate gene expression and inhibit cancer cell growth and proliferation to ultimately alleviate or even cure cancer. This field of research requires more corresponding experiments to improve the therapy choices for patients. In addition, i-motifs can function as DNA carriers or probe molecules, playing important roles in transmitting gene drugs in the nano-drug delivery system with the rapid development of nanomedicine.
